# *Montiphylax*, (Trichoptera, Limnephilidae), a new genus to accommodate the western North American species: *Stenophylaxantennatus* Banks, 1900, *Philocascathor* Nimmo, 1971, and *Philocascaalba* Nimmo, 1977

**DOI:** 10.3897/zookeys.845.31155

**Published:** 2019-05-15

**Authors:** David E. Ruiter, Robert A. Mutch

**Affiliations:** 1 235 SW Central Avenue, Grants Pass, Oregon 97526, USA Unaffiliated Grants Pass United States of America; 2 4- 309 Street SE, Medicine Hat, Alberta, Canada Unaffiliated Medicine Hat Canada

**Keywords:** Association, Cascade Mountains, diagnostic key, distribution, female, larvae, Rocky Mountains, systematics

## Abstract

*Montiphylax*, a new genus within the Limnephilidae, is proposed to include *Philocascaalba*, *P.antennata*, and *P.thor*. Characters for the adults and larvae are presented to separate *Montiphylax* from *Philocasca*. A summary of the distribution and life history of the *Montiphylax* species is provided. A key to ease separation of the known North American caddisfly larvae with gill clusters of single filaments is given.

## Introduction

The generic placement for the seven *Philocasca* taxa [*P.alba* Nimmo, 1977, *P.antennata* (Banks, 1900) as *Stenophylax* Kolenati, 1848, *P.banksi* (Denning, 1941) as *Anisogamus* McLachlan, 1874, *P.demita* Ross, 1941, *P.oron* Ross, 1949, *P.rivularis* Wiggins & Anderson, 1968, *P.thor* Nimmo, 1971] has been confused since Schmid (1955) placed *Stenophylaxantennatus* within *Philocasca*. Wiggins and Anderson (1968) examined the known *Philocasca* taxa at that time and accepted Schmid’s placement of *S.antennatus*in *Philocasca*. Two additional taxa (*P.thor* Nimmo, 1971, *P.alba* Nimmo, 1977) were subsequently described. Mutch (1981) pointed out the polyphyletic nature of *Philocasca*. Vshivkova et al. (2007) elevated *Philocasca* to the subfamily Philocascinae based only on the characters of *P.rivularis*. In order to clarify the status of *Stenophylaxantennatus*, a new genus, *Montiphylax* (Limnephilidae, Limnephilinae) is proposed to accept *P.antennata*, *P.thor*, and *P.alba*.

Wiggins and Anderson (1968) presented, in detail, the prior problems associated with description and relationships within the *Philocasca* and its placement within the Limnephilidae. These problems were the result of only five male specimens spread across the four known species. No females, or larvae, had been associated at the time. And, at the time, only Banks and Ross seem to have examined those same specimens, and they disagreed on their generic placement. Wiggins and Anderson (1968), with still only the type of *S.antennatus* male available, did not acknowledge the significant generic dissimilarities pointed out by Banks and added no further information to support their placement of *S.antennatus* within *Philocasca*. In the discussion below we present the detailed history of the confusion, and rationale for the creation of a new genus, *Montiphylax*, to resolve the confusion. The information is based, in part, on now available female and larval associations.

Also, since Wiggins and Anderson (1968), *Philocasca* has been removed from the Limnephilinae and placed within the Philocascinae (Vshivkova et al. 2007). *Philocasca* can be distinguished by many non-Limnephilinae characters: wings very broad, rounded throughout, without obvious color patterns/spotting, etc.; mesonotal warts absent; female without mesal lobe of vulvar scale; larvae with strongly rounded anterior portion of pronotum without transverse groove, and with unique, long, flattened, scale-like setae on head and pronotum. The arrangement and shape of these larval setae are diagnostic for the *Philocasca* species.

While the spotted wings, female three-part vulvar scale, and pronotal transverse groove of the larva currently place *Montiphylax* within the Limnephilinae sensu Vshivkova et al. (2007) its further placement is unclear. This has resulted in the need to clarify the larval characters currently used in North American larval keys (Wiggins 1996, Morse and Holzenthal 2008) to separate the genera. We have provided a key to more consistently separate these subfamilies and genera.

## Materials and methods

Abdomens were removed, cleared in 10% potassium hydroxide, mounted in glycerin and examined/imaged with the use of Leica stereo and Olympus compound microscopes, Canon DSLR cameras and EOS image capture software. Subsequent images were processed with Zerene image stacking software and Photoshop Elements image editing software. All collected material is preserved in 70% ethyl alcohol. Barcoding was successful for *M.antennatus* and Barcode of Life Datasystems, Ratnasingham and Hebert (2007), has *M.thor* barcodes available. Morphological terminology follows that of Wiggins (1996), Wiggins and Currie (2008) and Schmid (1998).

## Taxonomic accounts

### Diagnosis of the new genus *Montiphylax*

The *Montiphylax* male genitalia superficially resemble *Philocasca*; however, examination of head, setal warts, venation, and 9^th^ and 10^th^ segments shows no sister relationship. Vshivkova et al. (2007) completely separated *Philocasca* from the Limnephilinae based on characters of *P.rivularis*. *Philocasca* adults are readily separated from *Montiphylax* adults by the lack of irrorate forewings and metanotal setal warts in *Philocasca*. *Montiphylax* females have a mesal lobe on the vulvar scale that *Philocasca* females lack. *Montiphylax* larvae lack head and thoracic scale setae that are present on *Philocasca* larvae. *Montiphylax* larvae are distinguished from other North American Limnephilidae larvae by the combination of: pronotum not convex in lateral view, with a distinct indentation present between the anterior third to half of pronotum; all gill clusters consist of single filaments; sclerites of metanotal setal area 2 broadly separated; lateral line gills absent, and forked lamellae absent.

### Limnephilidae, Limnephilinae

#### Montiphylax gen. n.

Taxon classificationAnimaliaTrichopteraLimnephilidae

http://zoobank.org/BB66211A-F24F-46F8-B205-1ACEB1488D00

Figures 1 , 2 , 3 , 4 , 5 , 6 , 7 , 8 , 9 , 10

##### New combinations.

*Stenophylaxantennatus* Banks, 1900, new combination (type species); *Philocascaantennata* (Banks, 1900), new combination; *Philocascaalba* Nimmo, 1977, new combination; *Philocascathor* Nimmo, 1971, new combination

##### Description.

**Figure 1.**
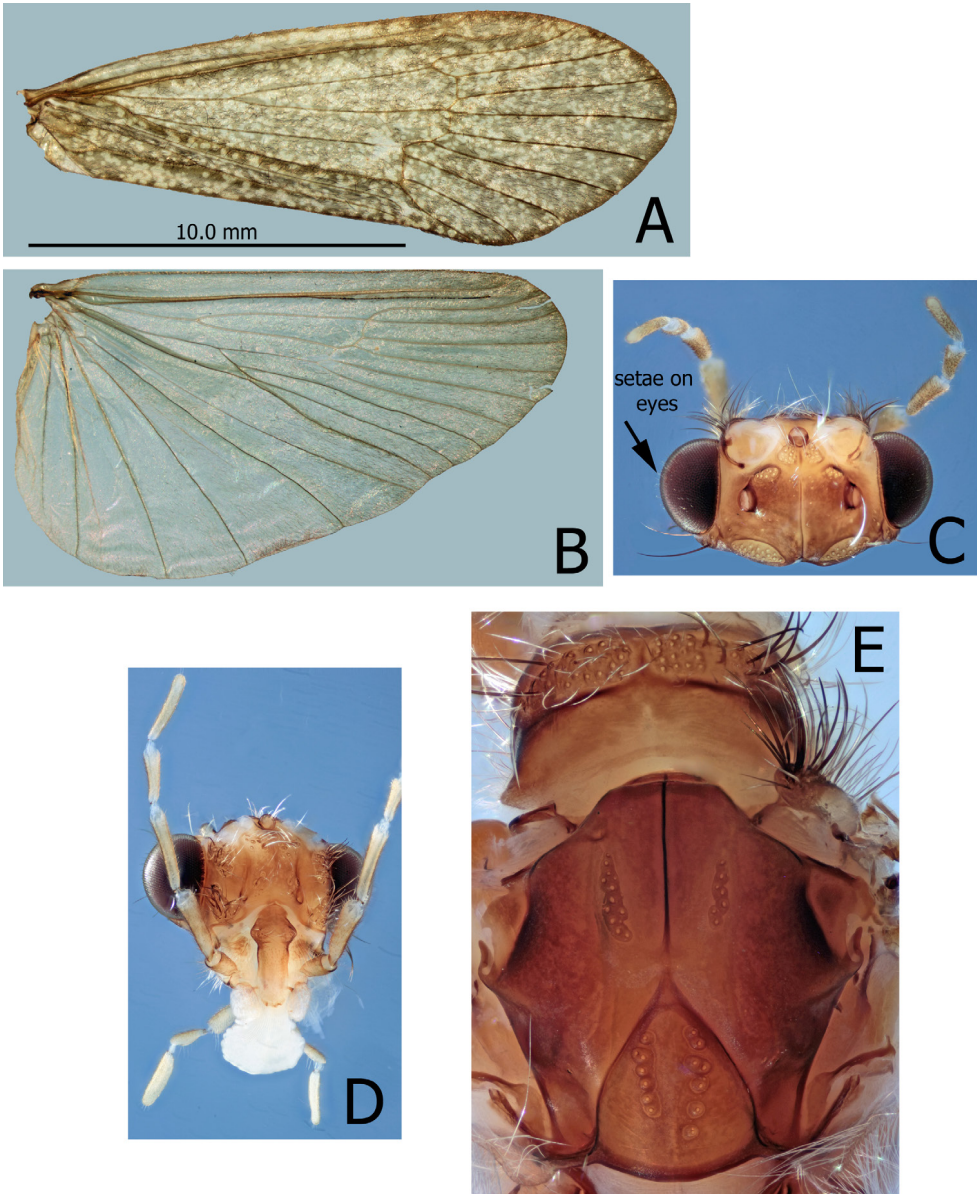
*Montiphylaxantennatus.* Male (**A–E**) **A** right side forewing, dorsal **B** right side hindwing, dorsal **C** head, dorsal **D** head, anterior **E** prothorax and mesothorax, dorsal.

Adult (Fig. 1). Head to wingtip length: *Montiphylaxantennatus* male - 19–21 mm (n = 5); *antennatus* female - 19–21 mm (n = 5). *Montiphylaxalbus* male - 18–19 mm (n = 8); *albus* female - 18–20 mm (n = 3). *Montiphylaxthor* male - 18–21mm (n = 3). Eyes large with numerous fine setae located between ommatidia. Head rectangular in dorsal view; ocelli large, posterior ocelli set midlength. Several small setae scattered on head surface, primarily between and behind posterior ocelli. Two pair of warts evenly spread between anterior and posterior ocelli. Posterior warts small, linear with acute apices. Malar projection as long as length of 1^st^ labial palp segment. Antennae shorter than wing length, stout, basal segment shorter than eye width.

Pronotal warts oval, wide, nearly touching mesally. Mesonotal macrosetae in linear row, basal warts located midlength, slightly merged but not fused into a single smooth oval wart; mesocutellar macro setae scattered linearly, less fused than mesonotal wart; most dorsal head and thoracic setae pale.

Forewings (in alcohol) reddish brown, finely irrorate; third radial vein and discoidal cell with a fairly long common boundary approximately equal to length of the first transverse vein; discoidal cell longer than its pedicel; all apical cells without pedicels. Anterior and posterior anastomosis nearly perpendicular to wing length, clearly located distad from posterior anastomosis. Hindwings brown, without irrorations; discoidal cell longer than its pedicel; all apical cells without pedicels. Anterior anastomosis perpendicular to wing length, clearly located distad from posterior anastomosis. Posterior anastomosis strongly oblique to wing margin. Most setae on wing membrane recumbent.

Legs long and thin, tarsi long with basal segment more than 0.5 length of mesotibia, Apical tarsal segments without ventral spines. Spines on legs black, spurs golden, spur count 1-2-4.

Male genitalia (Figs 6, 8, 9). Eighth segment without adornment, slightly swollen on dorsal apical margin. In lateral view 9^th^ segment annular, narrowed dorsally and broadest at base of short, tall inferior appendages; deeply invaginated dorsally in caudal view. Tenth segment cupped anteriorly, appearing as two, slightly fused hemispheres extending broadly anteriad within the 9^th^. Superior appendages of 10^th^ fused to intermediate appendages; in lateral view, variously broad and projecting, diagnostic by species. In dorsal view intermediate appendages of 10^th^ extended mesally as two long, tapered, downward directed, thin parallel projections. Inferior appendages of 10^th^ short, broad ventrally; nearly fused mesally. Phallus with heavily sclerotized, dorsomesal, conical projection at base of dorsolateral parameres. Parameres heavily sclerotized, projecting fingerlike, parallel to body of phallus; shape diagnostic by species. Apical portion of phallus membranous, extensile, with ejaculatory duct ending in an apical, upward directed, sclerotized cup.

Female genitalia (Figs 7, 10), female of *M.thor* unknown. Ninth tergite and sternite fused laterally; 10^th^ segment fused to 9^th^, broadly projecting distad to blunt, rounded apex in lateral view. In dorsal view 10^th^ apex cleft to approximately midlength mesally. In ventral view, 9^th^ sternal lateral projections fused to mesal supragenital plate. Vulvar scale stout, broadly merged to 8^th^ segment laterally, with wide, smoothly conical, medial lobe slightly shorter than lateral lobes.

**Figure 2.**
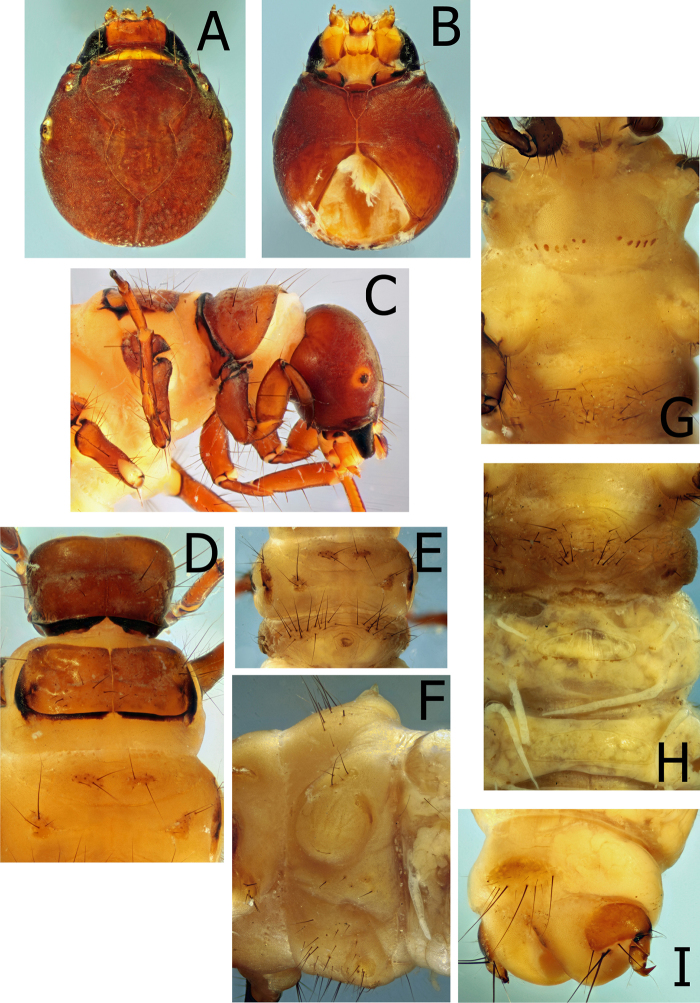
*Montiphylaxalbus*. Larva (**A–I**) **A** head, dorsal **B** head, ventral **C** head, pro & mesothorax, lateral **D** pro, meso & metathorax, dorsal **E** metathorax and 1^st^ abdominal segment, dorsal **F** lateral spacing hump, left lateral **G** pro, meso & metathorax, ventral **H** 1^st^, 2^nd^ and 3^rd^ abdominal sternites, ventral **I** 9^th^ tergite & anal prolegs, dorsal.

**Figure 3.**
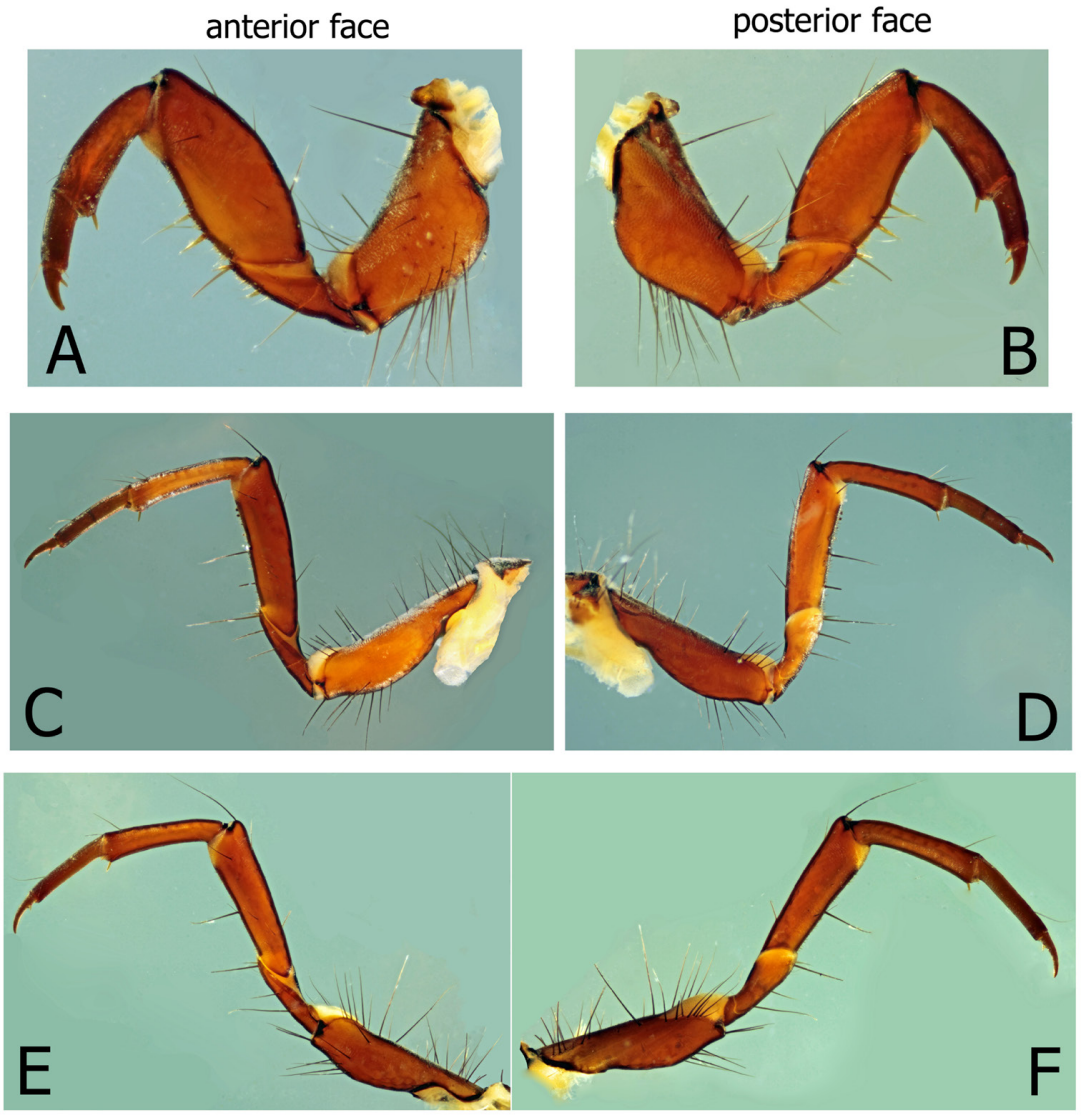
*Montiphylaxalbus*. Larval legs (**A–F**) **A** right side prothoracic leg, anterior face **B** right side prothoracic leg, posterior face **C** right side mesothoracic leg, anterior face **D** right side mesothoracic leg, posterior face **E** right side metathoracic leg, anterior face **F** right side metathoracic leg, posterior face.

**Figure 4.**
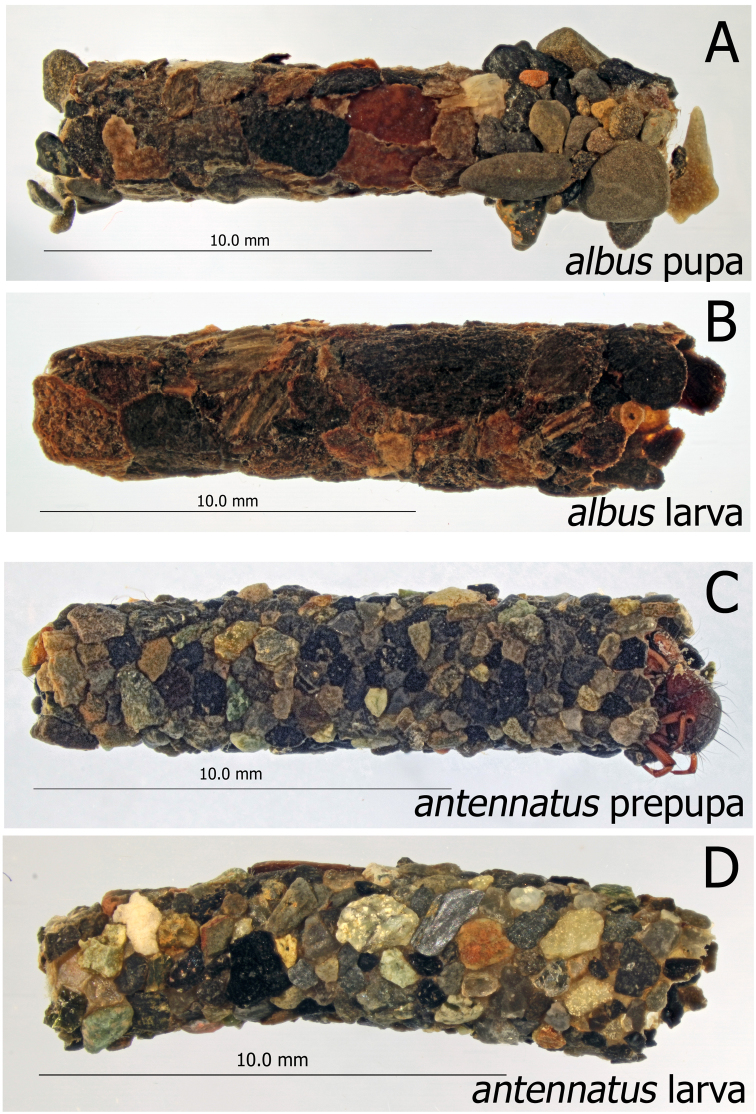
*Montiphylax*. Pupal and larval cases (**A–D**) **A***M.albus* pupa case, lateral **B***M.albus* larva case, lateral **C***M.antennatus* prepupa case, lateral **D***M.antennatus* larval case, lateral.

Larva (Figs 2–4), (n = 3 *antennatus* and 4 *albus*; larva of *M.thor* unknown). Head nearly circular in dorsal view, widest mid-length, rugulose, specimens in alcohol rust colored. Muscle scars indistinct. Typical limnephiloid setation, without accessory setae. Anterior ventral apotome small, equal, or shorter than ventral ecdysial suture. Submental sclerites distinctly separated from stipes and mesally separated.

Prothorax rust colored (in alcohol) like head; setae scattered, without distinct setal areas; in lateral view, slight transverse indentation approximately midlength; posterior margin thick and dark, with deep, preceding furrow; ventrally with a single, wide, short, prosternite between legs. Mesothorax paler than prothorax; setal areas nearly merged; posterior black margin extending along lateral margin nearly to anterior margin; ventral area with a linear row of small, dark, mesosclerites along each side of posterior margin. Dorsal metanotal setal areas on distinct sclerites, without additional setae on metonotal membrane. Ventrally, mesosternite with very pale linear row of small sclerites along each side of posterior margin, may be difficult to see, appearing as a row of indentations. Legs typical limnephiloid form with short, stout proleg and hind leg the longest. Meso and metafemur with two, long, dark setae ventrally and a row of very small, fine spinelike setae along entire ventral setal margin. Anterior face of meso and metafemur with numerous accessory setae; posterior face usually with one or two accessory setae.

First abdominal segment with numerous long setae anterior of dorsal spacing hump; lateral spacing hump with several small sclerites on dorsal margin and one larger sclerite along posterior margin; ventrally with numerous long setae and a pair of posterior setal warts on mature larvae. Chloride epithelia present ventromesally, much wider than long (*albus* 2–7 [n = 3]; *antennatus* 3–7 [n = 4]). Lateral fringe on segments 2–8 (*antennatus*), 3–8 (*albus*); segment 2 portion is very short. Forked lamellae absent. Gill clusters consist of single filaments dorsally on segment 2 through 5 or 6, ventrally on 2 through 5, lateral line gills absent.

Ninth tergite with 3–4 pairs of long setae and several additional shorter setae. Lateral sclerite of anal proleg without short, stout, pale setae. Anal claw with single dorsal accessory hook.

Pupal case of final instar slightly curved, non-tapered, wood and mineral, or all mineral construction. The limited larval material available indicates the earlier instars may build vegetation cases.

**Figure 5.**
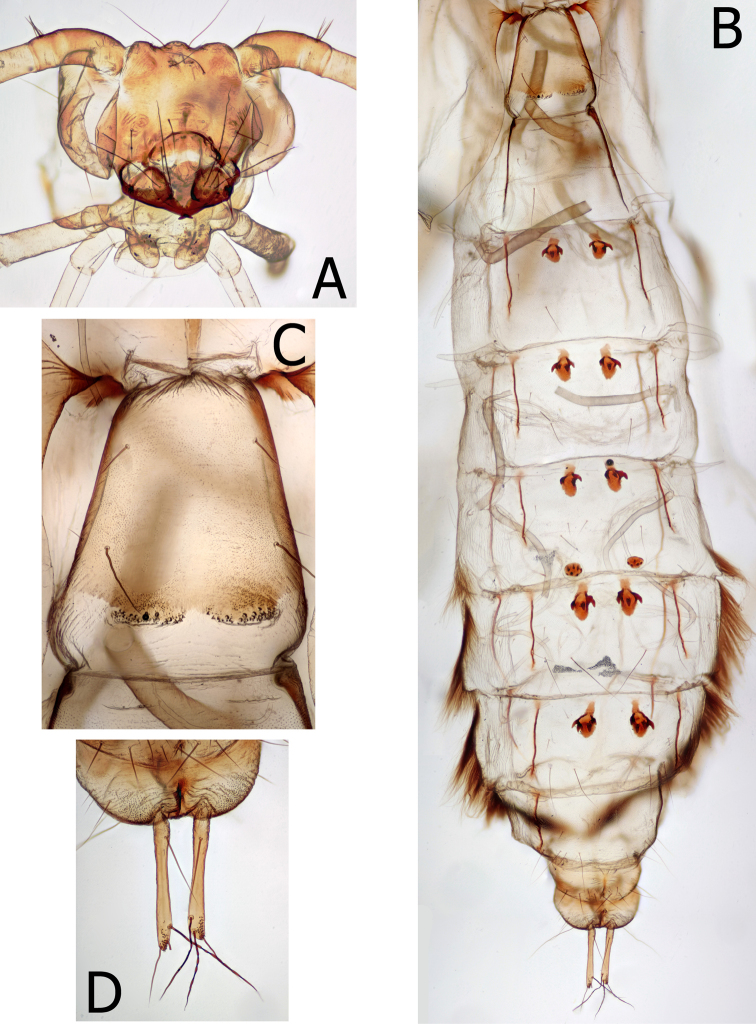
*Montiphylaxalbus*. Pupa (**A–D**) **A** head, anterior **B** abdomen, dorsal **C** 1^st^ abdominal segment, dorsal **D** last abdominal segment, dorsal.

**Pupa** (Fig. 5), (n = 4 *albus*). Labral setae long, with apices spirally twisted, not hooked. Mandibles triangular with apical half strongly tapered and flattened into an acute blade; mesal edge of blade slightly serrate. Antennal scape with a ventrolateral setal tuft and a single seta at the dorsal margin. Second antennal segment with a dorsal tuft. Antennae shorter than abdominal apex. Spined ridge of 1^st^ abdominal tergite weak, linear with small scattered spines. Anterior hook plates present on abdominal tergites 3–7, with strong hooks directed posteriad. Small, oval posterior hook plates present on tergum 5. Abdominal lateral fringe present on segments 5–8. Anal processes elongate, slender, tubular, with patch of minute, dorsal spines at apex. Four long setae on each anal process; one located basally, and three apically.

##### Etymology.

*Montiphylax* from the Latin *montis* (mountain) and the Greek *phylax* (guard), referring to the mountainous habitat of this genus.

##### Differential diagnosis of the Montiphylax species.

The male of *M.antennatus* is distinguished from *M.albus* and *M.thor* by the long, narrow superior appendages in lateral view and the blunt paramere apex. The *M.thor* superior appendage has a wide base, appearing more triangular in lateral view. The superior appendage of *M.albus* is shorter than either *M.antennatus* or *M.thor* although wide at the base like *M.thor*. In lateral view, the paramere apex of *M.albus* and *M.thor* is acute; in *M.albus* the paramere apex is downturned while it is upturned in *M.antennatus* and *M.thor*.

The female of *M.antennatus* is separated from *M.albus* by the round anterior margin of the vaginal apparatus that is more quadrate in *M.albus* in ventral view. The female of *M.thor* is unknown. The pupa of only *M.albus* is known.

The lateral setal fringe of *M.antennatus* larvae originates on the posterior margin of the 2^rd^ abdominal segment while the lateral fringe of *M.albus* starts at the anterior margin of the 3^rd^ segment. The larva of *M.thor* is unknown.

#### Montiphylax antennatus

Taxon classificationAnimaliaTrichopteraLimnephilidae

(Banks, 1900)

**Figure 6.**
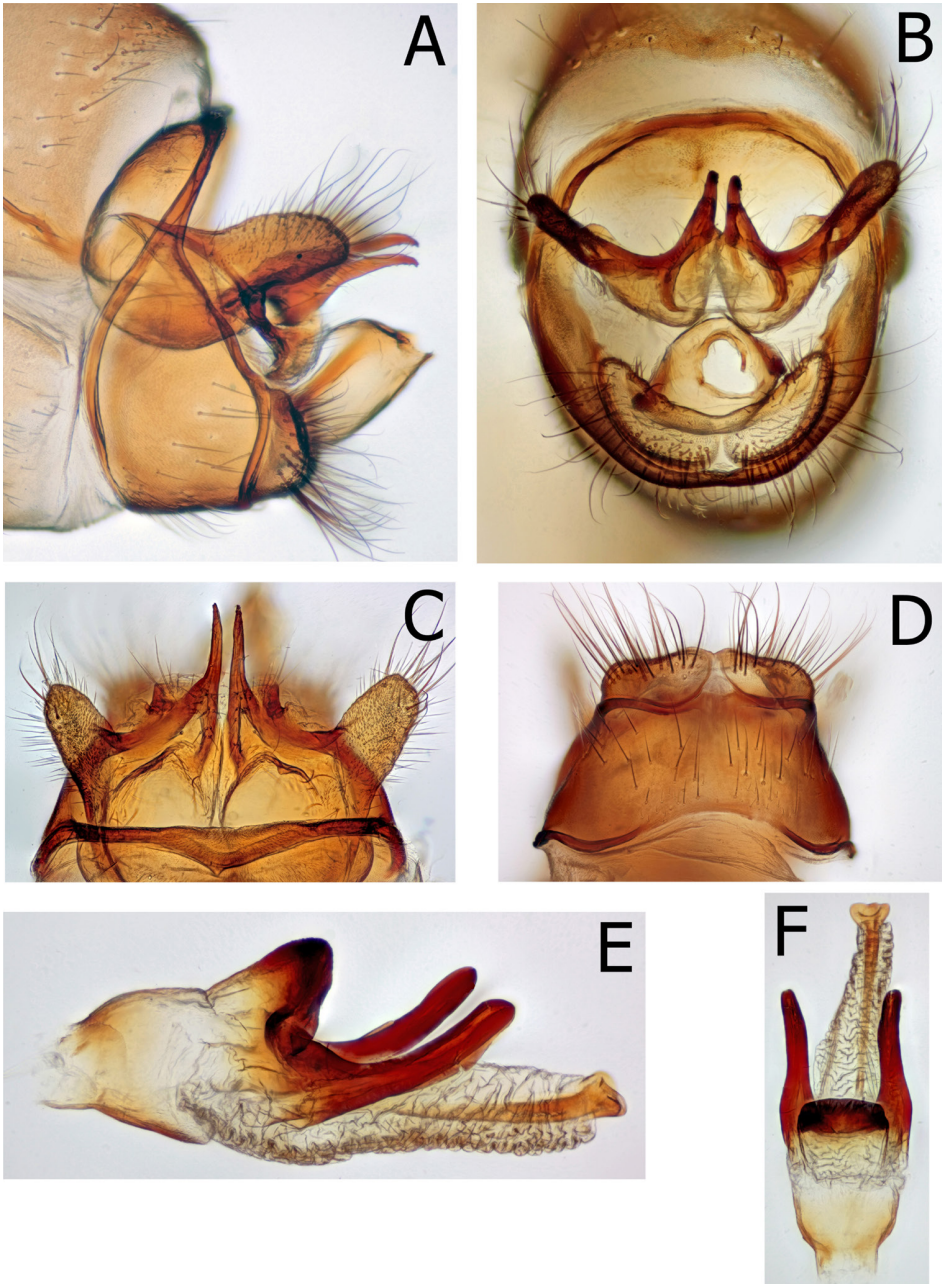
*Montiphylaxantennatus.* Male **(A–F) A** genitalia, left lateral **B** genitalia, caudal **C** genitalia, dorsal **D** genitalia, ventral **E** aedeagus, left lateral **F** aedeagus, dorsal.

**Figure 7.**
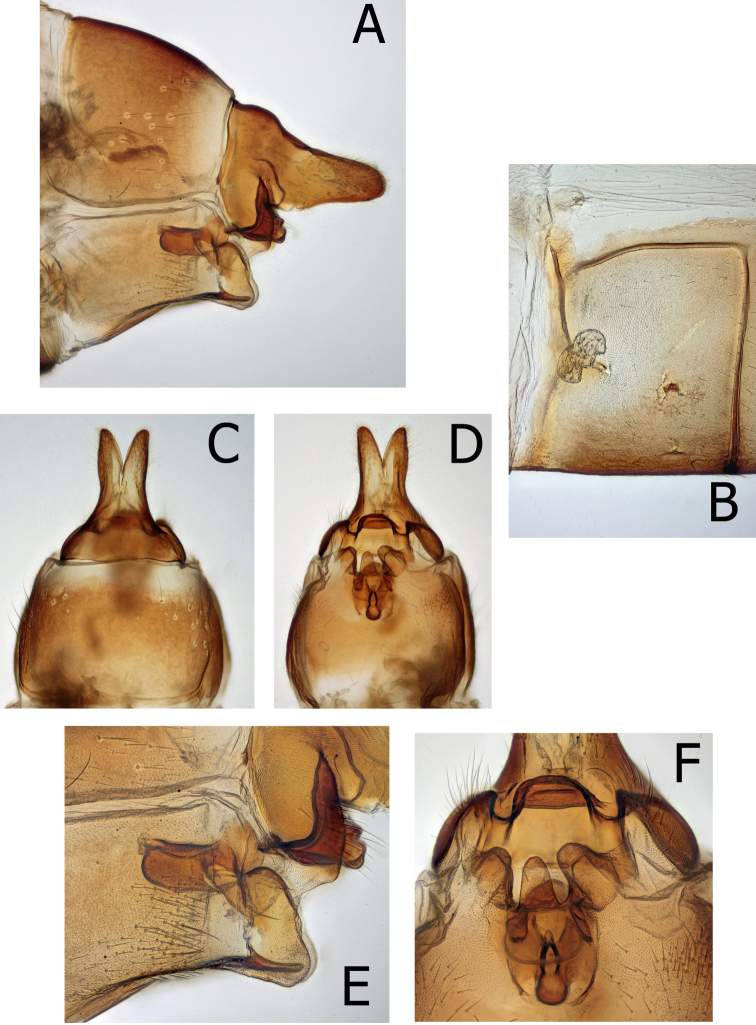
*Montiphylaxantennatus*. Female **(A–F) A** genitalia, left lateral **B** 5^th^ sternite, left lateral **C** genitalia, dorsal **D** genitalia, ventral **E** genitalia, left lateral **F** genitalia, ventral.

Figures 1 , 4C, D , 6 , 7

Stenophylax antennatus Banks, 1900: 254–255 (male description) Washington.
Anisogamus antennatus Milne, 1935: 29; Banks, 1943: 350, fig. 41 and 50 (describes 2^nd^ specimen from Wallace, Idaho. This Idaho specimen is M.thor, see discussion below.).
Stenophylax antennatus Ross, 1944: 299.
Philocasca antennata Schmid, 1955: 201; Flint, 1966: 379, fig. 2m, n; Wiggins & Anderson, 1968: 74; Wiggins, 1977: 274; Mutch, 1981: 223; Wiggins, 1996: 338; Schmid, 1998: 121; Ruiter et al., 2005: 162; Blinn & Ruiter, 2013: 292.

##### Description.

Head to wingtip length: male 19–21 mm (n = 5); female 19–21 mm (n = 5).

Male genitalia (Fig. 6): 8^th^ tergite without dorsal modified spines or projections. Ninth segment annular with tergite narrow and strap-like, directed distad dorsally resulting in slightly sigmoid anterior margin of 9^th^ in lateral view; remainder of 9^th^ broad in lateral view, directed downward at approximately a 45 degree angle from the narrow tergite; inferior appendages appearing separated from 9^th^. In caudal view, inferior appendages slightly cupped around phallic apparatus and slightly separated mesally. Tenth segment cupped anteriorly, appearing as two, slightly fused hemispheres extending broadly anteriad within the 9^th^. Superior appendage nearly twice as long as tall in lateral view. Intermediate appendages arise ventrally, strongly sclerotized and extending as two long, tapered, parallel projections, curving downward at apex. In caudal view, below the intermediate appendages are paired, curved, narrow projections nearly surrounding the anus. Phallus large, with strongly sclerotized phallicata; membranous endophallus with dorsal, strongly sclerotized band, projected upward and distad apically; strong, thick parameres originate at the base of the dorsal band, extend distally; tapering to blunt, rounded apex in lateral view and slight curved upward throughout. Parameres originate dorsolaterally and extend posteriorly over 3 times as long as wide; thick throughout, ending in blunt, rounded apex in dorsal view. The aedeagus apex a sclerotized tube within an extensile sheath; ending in the bottom of a posteriorly directed sclerotized cup.

Allotype female genitalia (Fig. 7): 9^th^ segment fused laterally and incomplete ventrally, separated ventrally by broad supragenital plate. Ninth fused with 10^th^; 9^th^ tergite ca. half as long as 10^th^ in lateral view; in lateral view 10^th^ with dorsal and ventral margins slightly tapered throughout to rounded apex. In dorsal view, 10^th^ cleft less than half the distance to base. In lateral view, ventrolateral corners of 9^th^ extend distally beyond remainder of 9^th^; acute at apex and directed inward in ventral view. The medial lobe of the vulval scale short, shorter than lateral lobes, longer than wide. The vaginal apparatus, in lateral view, rectangular, ca. twice as long as tall; in ventral view, egg-shaped with smoothly rounded anterior margin.

##### Material examined.

WA: Whatcom County, stream near Heather Meadow Information Center on Artist Point Road, D.W. Blinn, 28 July 2009, blacklight trap, 2M; stream on Fire and Ice Trail, near top of Mt. Baker Highway, 48.85586 –121.68795, D.W. Blinn, 13 August 2012, 2L, 1M, 1F; spring stream, ca. 150 feet southeast of outhouse at Mt. Baker Information Center parking lot, near top of Highway 542, 48.85378 –121.68543, D.E. Ruiter, 5 August 2012, 1L; meltwater stream on Fire and Ice trail, in Heather Meadows, Mt. Baker, D.W. Blinn, 31 July 2009, 13M, 3F (2M to National Museum of Natural History; 2M, 1F to S. Chuluunbat; 1M, 1F to Canadian National Collection, remainder in D.E. Ruiter personal collection); Terminal Lake on Mt. Baker Highway, D. W. Blinn, black light trap, 28 July 2009, 1M.

#### Montiphylax thor

Taxon classificationAnimaliaTrichopteraLimnephilidae

(Nimmo, 1971)

Figure 7 , 8

Philocasca thor Nimmo, 1971: 147–148, figs 143a, b, 545–547, 653.

##### Notes.

Nimmo (1971) described *M.thor* from a single specimen collected within Jasper National Park, Alberta. The Barcode of Life project has collected additional males from the Willmore Wilderness in Alberta, ca. 150 km northwest of the *M.thor* type locality. This Barcode of Life material was compared to the *M.thor* holotype and found to be the same. To date, females or larvae of *M.thor* have not been located.

Wiggins and Anderson (1968) mentioned the dark ventral surface of the *M.antennatus* scape described by Banks (1900) was absent on the Idaho *M.antennatus* specimen that Banks (1943) described, implying it was present on the *M.antennatus* type. The darker ventral surface of the antennal scape is present on the Mt. Baker *M.antennatus* males and females examined here. However, it is absent on our *M.albus* material, that have a concolorous scape. *Montiphylaxthor* males have a darkened scape with the ventral surface pale - the exact opposite coloration pattern from *M.antennatus*. The Banks (1943) Idaho specimen was examined and it has the light ventral surface of the scape and upturned, acute parameres of *M.thor*. Neither Banks (1943) nor Wiggins and Anderson (1968) recognized this Idaho specimen as a new species. Our genetic comparisons of *M.antennatus* and *M.thor* also support Nimmo’s conclusion that *M.thor* is a valid species. Our determination that the Banks (1943) Wallace, Idaho, record represents *M.thor* extends the *M.thor* range another 600km farther south along the Rocky Mountains. This also results in *M.thor* occurring both further north and south than *M.albus* along the Rockies. Wallace, Idaho, is a historic silver mining city in the South Fork of the Coeur d’Alene River Valley. It is likely the *M.thor* specimen was collected at higher elevations in the surrounding area.

##### Description.

**Figure 8.**
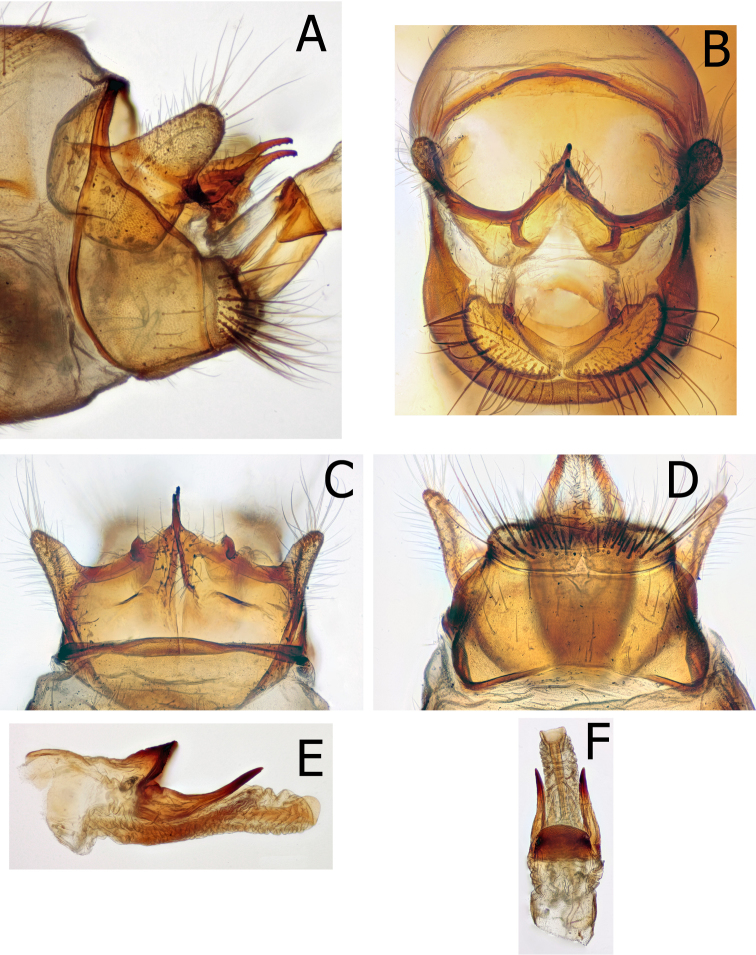
*Montiphylaxthor*. Male **(A–F) A** genitalia, left lateral **B** genitalia, caudal **C** genitalia, dorsal **D** genitalia, ventral **E** aedeagus, left lateral **F** aedeagus, dorsal.

Male genitalia (Fig. 8): The dark, conical, ventral sclerite of the phallic apparatus illustrated by Nimmo (1971) is absent from the holotype and the other *M.thor* specimens examined in this study. It is unknown what the Nimmo (1971) dark ventral sclerite represents. Eighth tergite without dorsal modified spines or projections. Ninth segment annular with tergite narrow and strap-like; remainder of 9^th^ broad in lateral view, directed posteroventrad at ca. a 45 degree angle from the narrow tergite, with tall, narrow inferior appendages nearly fused to 9^th^. In caudal view, inferior appendages slightly cupped around phallic apparatus and slightly separated mesally. Tenth segment cupped anteriorly, appearing as two hemispheres extending broadly anteriad within the 9^th^. Tenth segment appears fused mesally along anterior margin. Superior appendages approximately as long as tall, triangular in lateral view directed upward. Intermediate appendages arise ventrally, strongly sclerotized and extending as two long, tapered, parallel projections, curving downward at apex. In posterior view, below the intermediate appendages are paired, curved, narrow projections nearly surrounding the anus. Phallus large, with strongly sclerotized phallicata; membranous endophallus with dorsal, strongly sclerotized band, projected upward and distad apically; strong, thick parameres originate at the base of the dorsal band, extend distally; tapered to acute apex in lateral view and slightly curved upward throughout. Parameres originate dorsolaterally and extend posteriorly ca. 3 times as long as wide; tapering evenly throughout to acute apex in dorsal view. Aedeagal apex a sclerotized tube within an extensile sheath; ending in the bottom of an upward directed sclerotized cup.

##### Material examined.

Holotype male - Canadian National Collection Type # 10,588, Alpine Meadows, east of Mt. Edith Cavell, Jasper National Park, Alberta, habitat: Mountain Tarn, A. P. Nimmo, 10–12am, 4 July 1975; Alberta, Willmore Wilderness, 53.6858, -119.419, Hilchie & MacAuley, 21 July 2007, 3 males (Bold # 10ABCAD-004, 10ABCAD-006, & 10ANCAD-007).

#### Montiphylax albus

Taxon classificationAnimaliaTrichopteraLimnephilidae

(Nimmo, 1977)

**Figure 9.**
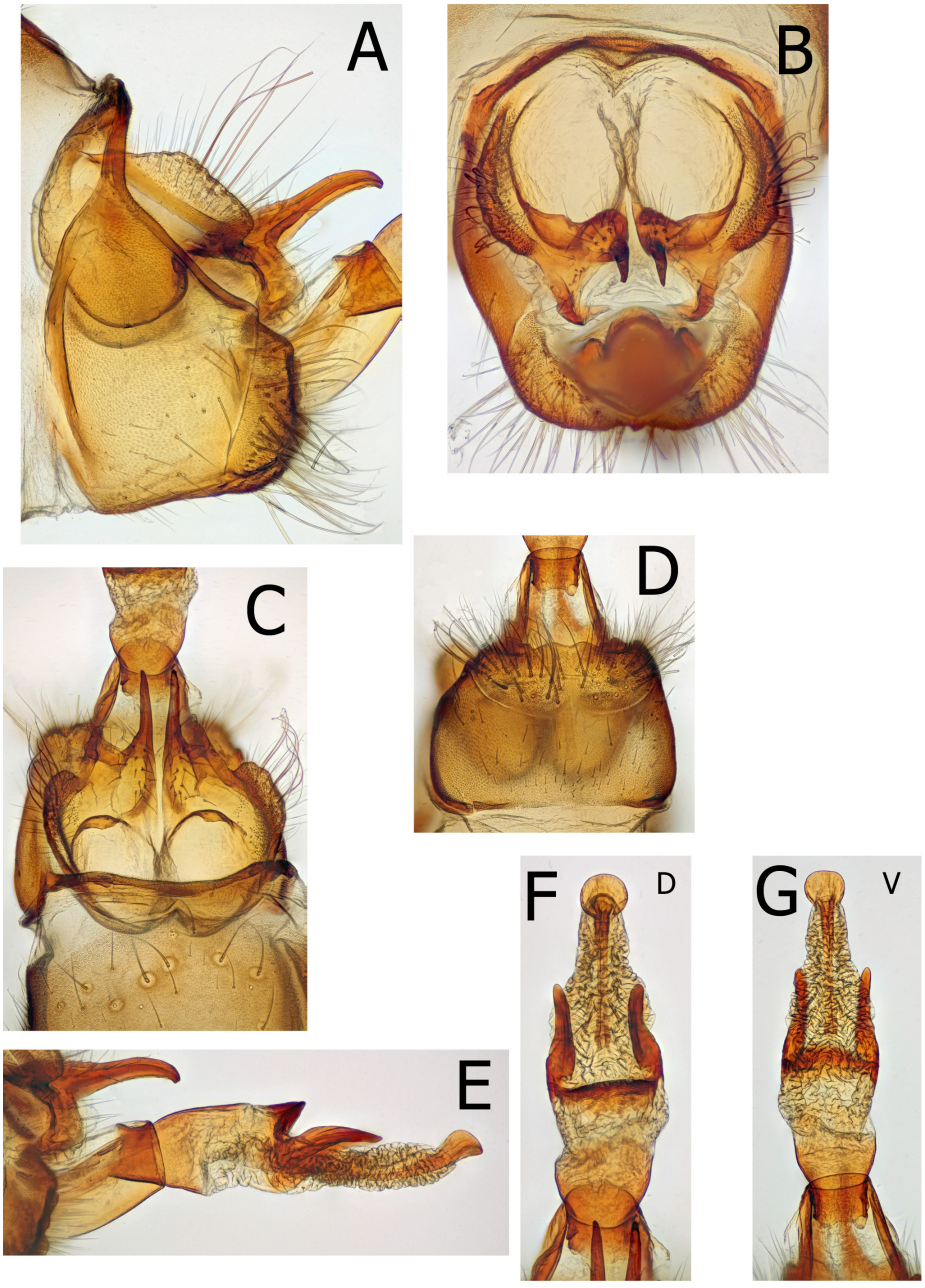
*Montiphylaxalbus*. Male **(A–G) A** genitalia, left lateral **B** genitalia, caudal **C** genitalia, dorsal **D** genitalia, ventral **E** aedeagus, left lateral **F** aedeagus, dorsal **G** aedeagus, ventral.

**Figure 10.**
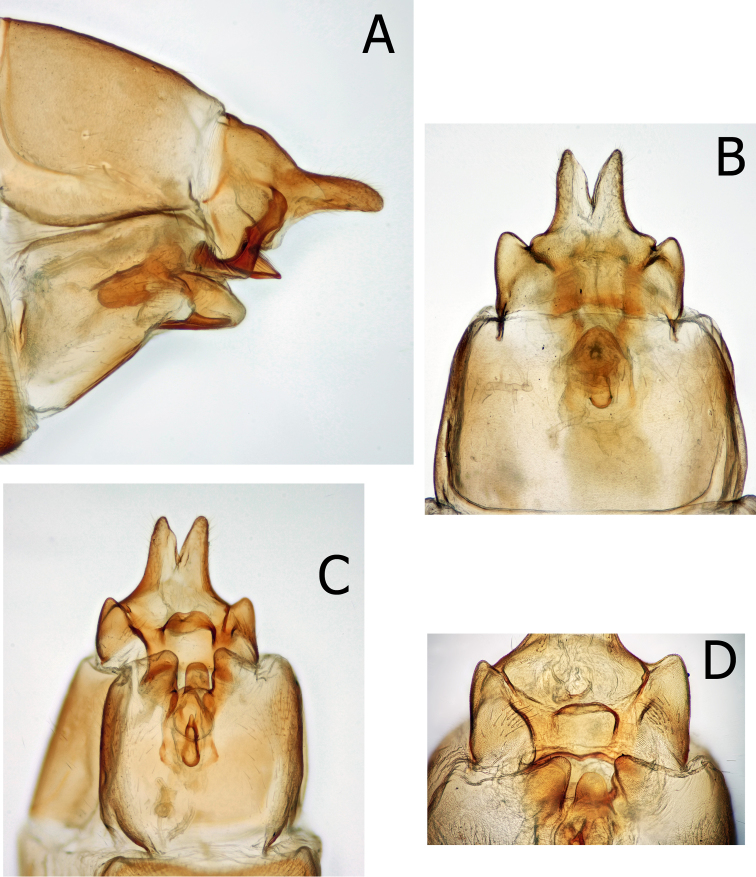
*Montiphylaxalbus*. Female **(A–D) A** genitalia, left lateral **B** genitalia, dorsal **C** genitalia, ventral **D** genitalia, ventral.

Figs 2 , 3 , 4A, B , 5 , 9 , 10

Philocasca alba Nimmo, 1977: 45–46, 49, figs 102–105, 121; Mutch, 1981: 222–228, fig. 43.

##### Notes.

Nimmo (1977) described *M.albus* and included characters to separate *M.albus* from *M.thor*. The second author reared from associated pupae (Alberta, Middle Fork Creek, Marmot Creek Experimental watershed, 1800 meters altitude, 50.94820 –115.15151) the male and female of *M.albus*, and provided figures of the male, female, and pupa. These are from the same watershed as the Nimmo paratype collection. Based on this *M.albus* material, we add descriptions and images for the *M.albus* adults, pupa and larva.

##### Description.

Head to wingtip length: male - 18–19 mm (n = 8); female - 18–20 mm (n = 3). General description of adults, pupa, and larva contained in genus description above.

Male genitalia (Fig. 9): 8^th^ tergite without dorsal modified spines or projections. Ninth annular with tergite narrow and strap-like; remainder of 9^th^ broad in lateral view; with tall, narrow inferior appendages nearly fused to 9^th^. In caudal view, inferior appendages curved around margin of 9^th^ base to meson of 9^th^, appearing fused mesoventrally. Tenth segment cupped anteriorly, appearing as two hemispheres extending broadly anteriad within the 9^th^. Tenth segment appears split mesally. Superior appendages taller than long in lateral view, directed downward and distad at ventral margin. Intermediate appendages arise ventrally, strongly sclerotized and extending as two long, tapered, parallel projections, curving downward at apex. In caudal view, ventrad to the intermediate appendages paired, curved, narrow projections nearly surround the anus. Phallus large, phallicata strongly sclerotized; endophallus with strongly sclerotized band dorsally, projected upward and distad apically; strong, thick parameres originate at the base of the dorsal band, extend distally, tapered to acute apex in lateral view and slightly curved downward throughout. In dorsal view, parameres originate dorsolaterally and extend posteriorly ca. 3 times width, tapering evenly to bluntly curved apex. Aedeagus apex a sclerotized tube within an extensile sheath, ending in the bottom of an upward directed sclerotized cup.

Allotype female genitalia (Fig. 10): 9^th^ segment fused laterally and incomplete ventrally. In lateral view, 9^th^ fused with, and nearly as long as 10^th^. Tenth with dorsal and ventral margins parallel throughout most of its length until rounded apex. In dorsal view 10^th^ cleft to approximately midlength. In lateral view, ventrolateral corners of 9^th^ clearly shorter than remainder of 9^th^; in ventral view, broadly rounded at apex and directed outward. Medial lobe of the vulval scale shorter than lateral lobes, approximately as wide as long. Vaginal apparatus rectangular in lateral view, ca. twice as long as tall; in ventral view, rectangular with anterolateral corners projecting outward.

##### Material examined.

Holotype male: Canadian National Collection # 151765, Rowe Brook, near Lower Rowe Lake, 6350’, Waterton National Park, Alberta, 12 June 1975, D.B. Donald [near 49.054706, -114.052434]; Alberta, Twin Creek, Marmot Creek Experimental watershed, 1800 meters elevation, R. Mutch, 1978, 4L, 50.94820 -115.15151; light trap, 1 July 1979 4M4F; 16 April 1980, 1 pupa reared to male; 16 April 1980 1 female pupa; 26 April 1980, 1 larva reared to female; 26 April 1980, 1 larva reared to male; 1 May 1980, 1 larva reared to male.

### Distribution and life history

Like *Philocasca*, all species of *Montiphylax* are known only in the Western Cordillera of North America. *Montiphylax* is the more northern of the two genera and has been found in locations ranging in latitude from ca. 46 to 54 degrees north. *Montiphylax* is known from the Cascade Range of Washington (*M.antennatus*), the Shoshone Range of Idaho (*M.thor*), the Selkirk Range of British Columbia (*M.thor*) and the eastern slopes of the Canadian Rocky Mountains in Alberta (*M.thor* & *M.albus*).

Adults of the three species of *Montiphylax* have been found at altitudes ranging from 1354m (4,442’) in the Cascade Mountains *(M.antennatus* Whatcom Co. Washington) to 1936m (6,350’) in the Rocky Mountains (*M.thor* Waterton National Park). The larvae of *M.antennatus* and *M.albus* inhabit small cold sub-alpine streams. The larva of *M.thor* is unknown but given the locations the adults have been taken, it seems highly likely the larvae of this species will be found in high mountain streams.

A detailed life history is known only for *M.albus*. To date this species has only been associated with small, cold sub-alpine mountain streams in the Eastern Slope of the Rocky Mountains of Canada. In these streams the life cycle is three years but the presence of a few intermediate sized larvae suggests that the life cycle is flexible. There are five instars. All instars build cases of detritus, predominately of cone bracts, bark, small bits of conifer needles and wood. Instars I – IV have a case with a non-curved triangular cross-section. The V instar case is cylindrical in cross section and is made principally of the same materials as earlier instars except that some small stones are added. The emergence period is from mid-May until mid-June and the flight period lasts until the last week of July. Egg masses have not been located in the field. Growth of larvae is confined to the ice-free period (June – early November). The larvae are detritivores but moss may play a significant role in the diet of the V instars (Mutch 1981).

The flight periods of *M.thor* and *M.antennatus* are not known but adults of *M.thor* have been collected in July and adults of *M.antennatus* collected in June/July/early August.

Final instar larvae of *M.antennatus* build a case that has more mineral content than that of *M.albus*.

## Discussion

*Montiphylax* forms a closely allied group with *Homophylax*. *Montiphylax*, *Chyranda*, *Clostoeca*, *Homophylax*, and *Phanocelia* all fall within the Limnephilidae Genera *Incertae Sedis* A of Vshivkova et al. (2007), and they all lack larval forked lateral lamellae. This lack of lateral lamellae is a rare condition within the Limnephilidae. During preparation of the enclosed key we also noted that larvae of *Pycnopsychegentilis* group also lack the larval forked lateral lamellae. Adult *Montiphylax* will track to couplet 21 in Schmid (1998) and then fail, not matching either choice. In Ruiter (2000)*Montiphylax* tracks to *Chyranda*. *Montiphylax* can be readily separated from *Chyranda* by the presence of irrorate wings in *Montiphylax*. *Montiphylax* larvae will track to *Homophylax* or *Philocasca* in Wiggins (1996) depending on how strongly sclerotized the lateral hump sclerites are. In Morse and Holzenthal (2008)*Montiphylax* will key to *Homophylax*, *Pycnopsyche*, or *Psychoglypha* for the same reason. The problem with current larval keys is that the arrangement and size of sclerites around the lateral spacing hump is more variable than presented in the keys. As more larval specimens of these western taxa are examined it has become obvious that the lateral hump sclerite spacing and size are not consistent within the genera, particularly within *Psychoglypha*. We have provided a new key for the North American Limnephilidae larvae that possess gill clusters comprised of only a single filament. Hopefully this will provide for more consistent results within these taxa and lead to improved larval species associations. The pupae of *Montiphylax* we have will not key to the limnephiloids in Wiggins and Currie (2008) because of the non-hooked labral setae. It is possible that all our specimens (n = 4) possess broken setae since these setae are often broken on pupal material. Since caddis pupae are still poorly described, further work at the family level will be needed.

### Key to the North American Limnephilidae larvae which possess gill clusters consisting of a single filament^1^

**Table:** 

1	In lateral view pronotum distinctly inflated at midlength (Wiggins 1996: fig.20.31)	**2**
‒	In lateral view pronotum not inflated, with transverse furrow in anterior half (Wiggins 1996: fig.20.34)	**3**
2	Large flattened scale-hairs along anterior margin of pronotum (Wiggins 1996: fig. 20.31)	**Philocascinae: *Philocasca***
‒	Pronotal scale hairs absent, margins of head pebbled, without carina, (Wiggins 1996: fig. 20.13)	*** Ecclisocosmoecus ***
3	Metanotal setal area 1 & setal area 2 sclerites large in relation to metanotum, distance between setal area 2 sclerites less than 3 times width of one setal area 2 sclerite, usually much less (Wiggins 1996: fig. 20.14)	*** Ecclisomyia ***
‒	Metanotal sclerites small, distance between setal area 2 sclerites greater than 3 times width of setal area 2 sclerite (Wiggins 1996: fig. 20.7)	**4**
4	Abdominal lateral lamellae absent (Wiggins 1996: fig. 20.7)	**5**
‒	Abdominal lateral lamellae present (Wiggins 1996: fig. 20.11)	**10**
5	Lateral line gills absent (Wiggins 1996: fig. 20.14)	*** Montiphylax ***
‒	Lateral line gills present (Wiggins 1996: fig. 20.7)	**6**
6	Setae present on metatergal membrane between setal area 2 sclerites (Wiggins 1996: fig. 20.11)	***Pycnopsyche* in part (*gentilis* group)**
‒	Setae absent on metatergal membrane between setal area 2 sclerites (Wiggins 1996: fig. 20.9)	**7**
7	Strong, pale, spines absent on anal proleg sclerite (Wiggins 1996: fig. 20.9)	**8**
‒	Pale spines present on anal proleg sclerite (Wiggins 1996: fig. 20.22)	**9**
8	Dorsal and ventral lateral gills present (Wiggins 1996: fig. 20.9)	*** Clostoeca ***
‒	Only ventral lateral gills present (Wiggins 1996:fig.20.7)	*** Chyranda ***
9	Meso and metafemora with numerous major ventral setae, stout, pale spines absent on 9^th^ tergal sclerite (Wiggins 1996: fig. 20.29)	*** Phanocelia ***
‒	Meso and metafemora with only two major ventral setae, stout, pale spines present on 9^th^ tergal sclerite (Wiggins 1996: fig. 20.22)	*** Homophylax ***
10	Basal segment of hind trochanter with more than 1 seta along the ventral surface (image 11A this paper)	**11**
‒	Basal segment of hind trochanter with only 1 seta on ventral margin, located at distal margin near suture (image 11B this paper)	**12**
11	Setae between metanotal setal area 2 sclerites in a straight line near posterior margin of segment (Wiggins 1996: fig. 20.33)	*** Pseudostenophylax ***
‒	Setae scattered near middle of metanotal segment between setal area 2 sclerites (Wiggins 1996: fig. 20.11)	*** Desmona ***
12	Lateral hump of abdominal segment 1 without sclerites near base of hump (Wiggins 1996: fig. 20.19)	**13**
‒	Lateral hump of abdominal segment 1 with sclerites near base of hump (Wiggins 1996: fig. 20.23)	**14**
13	Head and pronotum covered with small spines (Wiggins 1996: fig. 20.19)	*** Grensia ***
‒	Head and pronotum without small spines (Wiggins 1996: fig. 20.6)	*** Chilostigma ***
14	Abdominal lateral spacing hump with several small sclerites variously positioned near base (Wiggins 1996: fig. 20.34)	*** Psychoglypha ***
‒	Lateral spacing hump with a single, large sclerite along posterior margin (Wiggins 1996: fig. 20.36)	**15**
15	Mesonotal setal area 1 sclerites fused mesally (Wiggins 1996: fig. 20.23)	*** Hydatophylax ***
‒	Mesonotal setal area 1 distinctly separated (Wiggins 1996: fig. 20.36)	***Pycnopsyche* in part**

### History of *Philocasca* and *Montiphylax* systematic placement

Banks (1900) described *Stenophylaxantennatus* from a Mt. Rainier, Washington, specimen. At the time he pointed out it was not a true *Stenophylax* but was waiting for more specimens with which to describe a new genus. *Stenophylaxantennatus* was not mentioned again until the major review of the Limnephilidae (Milne 1935) where Milne placed *S.antennatus* within *Anisogamus* based on the forewing shape with long apical cells.

**Figure 11.**
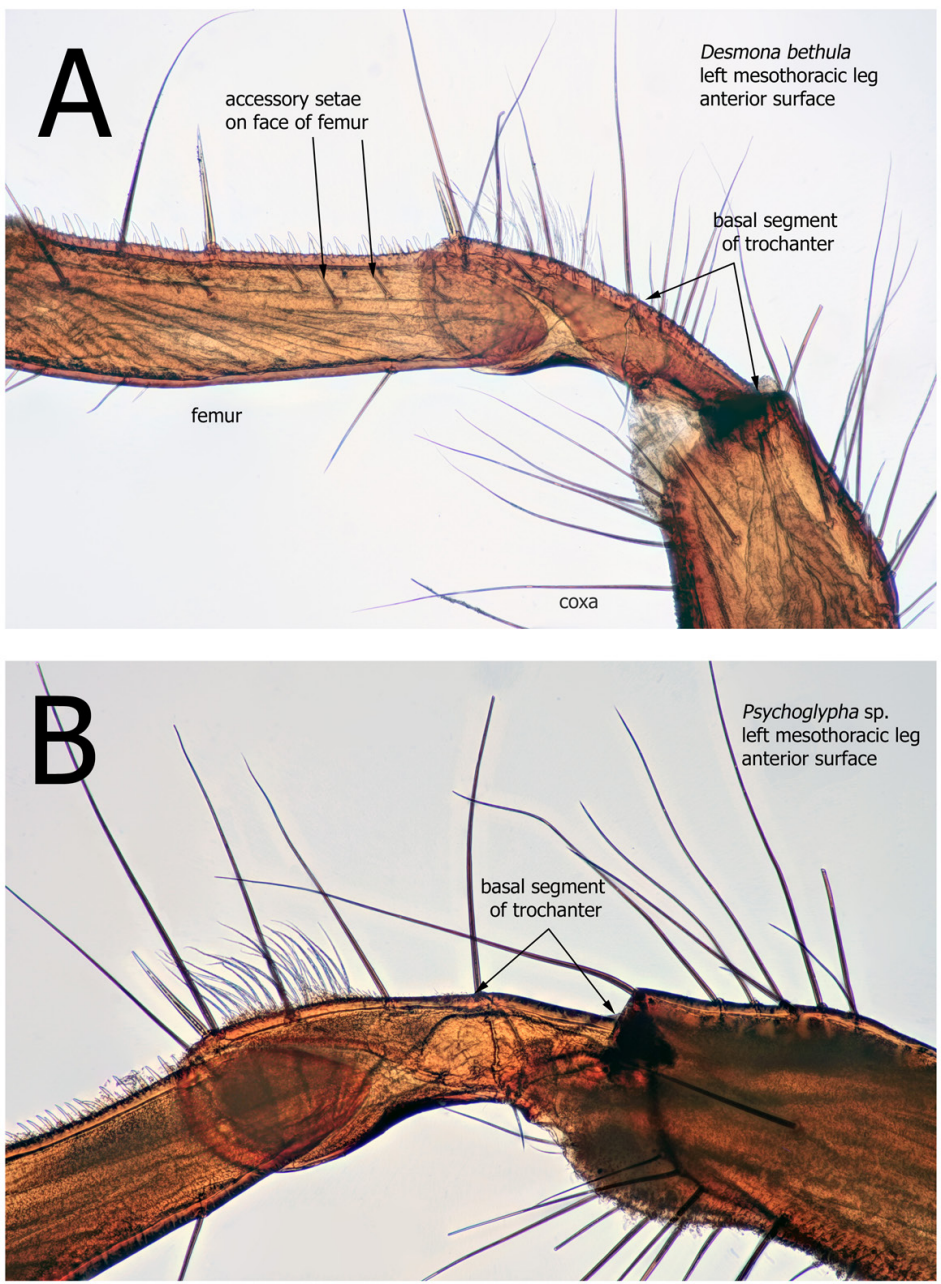
Mesothoracic trochanter. Larvae **(A, B) A***Desmonabethula*, left side, anterior surface **B***Psychoglypha* species, left side, anterior surface.

Denning (1941) described *Anisogamusbanksi* from a single Wallace, Idaho, male and mentioned that Banks (in lit.) thought it was near *Pseudostenophylaxedwardsi*. Denning’s *A.banksi* type information is: male, Wallace Idaho, 29 April 1938, Otto Huellemann; deposited at University of Minnesota collection. This locality and collector are the same as mentioned by Banks (1943), for a specimen of *S.antennatus*, although the date is different. Denning’s (1941) aedeagal figure (Fig. 11a) is upside down. The Denning holotype was examined during this study, is pinned and in good condition with the cleared abdomen and separated aedeagus within a separate microvial on the pin.

Ross (1941) created the genus *Philocasca* for a single male (*P.demita*) and placed it within the Limnephilidae, Limnephilinae, Stenophylacini. This placement was based on his conclusion of a close association between *P.demita* and *Anisogamus*, and followed Milne’s (1935) conclusion. [Until the recent description of a second species (Graf et al. 2015), *Anisogamus* was a monospecific genus based on *A.difformis* (McLachlan 1867) known from only a small area in Europe and thought to be most closely related to *Stenophylax*.] The *P.demita* type information is: Boyer, Oregon, Hood Craven Cabin, September 30, 1933, J.A.A.

The Boyer type locality is questionable as Hood Craven Cabin is not at Boyer. By 1933, Boyer, Oregon, had been relocated from the original locality in Lincoln County to its current location along Highway 18 (Benton County Museum 1981) in far southeast Tillamook County (45.06990 –123.72656) at ca. 600 feet altitude. Ross (1941) described both *Limnephilusectus* and *Rhyacophilaecosa* from Boyer that were collected 6 May 1934 and 15 July 1934 respectively by M.L.H. The identity of M.L.H. is unknown to the authors. These *L.ectus* and *R.ecosa* descriptions did not include Hood Craven Cabin as part of the type locality. In a summary of Oregon Tipuliidae collection records, Alexander (1954) indicates Hood Craven Cabin is the same as Saddle Mountain, Lincoln County, at 3,000 feet. Saddle Mountain is actually 2,220 feet elevation, and located ca. 60 miles SSW of Boyer. Wiggins and Anderson (1968) indicated that Boyer is in Lincoln County, possibly following the Alexander information, or the original post office location. It is likely an error was introduced by connecting Hood Craven Cabin with Boyer.

Banks (1943) indicated that the type of *Stenophylaxantennatus* Banks was a male and provided a re-description and clear, representative figures. He also indicated he had another male from Wallace, Idaho, 12 June (Huellemann) - the same locality/collector (although different date) as Denning’s *Anisogamusbanksi*. Banks (1943) did not mention Denning’s (1941)*Anisogamusbanksi* description, or where he thought *S.antennatus* should be placed.

Banks (1943), in his following description of *Drusinusfrontalis* [moved to *Eocosmoecus* Wiggins and Richardson, 1989], has a note indicating: “We have no species closely congeneric with *Anisogamus*, and [*Anisogamus*] *edwardsi* and [*Anisogamus*] *atripennis* are better in *Drusinus*”. [*Drusinus*Betten (1934) is a synonym of *Pseudostenophylax*; see Schmid (1955).] Then Banks (1943), in a discussion focused on *Philocasca*, disagreed with Ross’ conclusion that *Philocasca* was close to *Anisogamus* and proposed *P.demita* may be a member of *Phacopteryx*Kolenati (1848) [currently a synonym of *Anabolia* Stephens (1837); see Betten and Mosely 1940, Schmid 1950]. Banks also stated that Milne’s placement of *Anisogamusdisjuncta*, and *Asynarchuscostalis* as *Anisogamus* was incorrect, resulting in Banks’ conclusion that no North American taxa belonged within *Anisogamus*. In the following paragraph, Banks (1943) created the genus *Clostoeca* for *Clostoecasperryae* and moved *Anisogamusdisjuncta* to *Clostoeca*. It is clear that Banks had concluded *S.antennatus* did not belong within *Philocasca* Ross.

Ross (1944), without comment, moved *Anisogamusbanksi* to *Philocasca* along with *P.demita* and left *S.antennatus* as *Stenophylax*, apparently agreeing with Banks (1943).

Ross (1949) described *Philocascaoron* and pointed out (although did not illustrate) the aedeagal structure was typical for the genus. The primary characters Ross listed to separate *P.oron* from *P.demita* were width and thickness of mesal cerci processes (thin and curved ventrad) and reduced spur count (1-2-2). The *P.oron* type locality is Bear Creek, Clatsop County, Oregon, 12 April 1947, S.G. Jewett, Jr. [likely near 45.78172 -123.43585 C. Kerst and R. Wisseman pers. comm. 2018]. Wiggins and Anderson (1968) noted the collection of a 1-3-4 spur count *P.oron* specimen. During research for this paper, DER has also noted inconsistent spur counts in *P.demita* and *P.rivularis*. Often one hind leg preapical spur is totally absent while the second preapical spur is reduced to a nub. We have been unable to locate fresh Clatsop County, Oregon, *Philocasca* specimens for DNA analysis. The male and female differences presented to separate *P.oron* and *P.demita* are minor variations in shape of a couple structures. A larva distinguishable from *P.demita* has not been found, although the larvae of the other *Philocasca* species are readily separated. We suspect *P.oron* is a synonym of *P.demita*. The type locality is in an area that has been extensively clear-cut and developed for residential/recreational housing over the intervening years (C Kerst, pers. comm. 2018).

Schmid (1955) was the first to formally move *Stenophylaxantennatus* to *Philocasca*. It is not clear why Schmid did this as he indicated he had not examined any of the Ross *Philocasca* taxa and that the *S.antennatus* male was admittedly different from the rest of the *Philocasca*. Schmid also indicated that females, that would have improved his decision, were not available to him. Schmid (1955), in the discussion of *Stenophylax*, referenced a paper (Schmid 1955 in press) where he reviewed the genus *Stenophylax*. The Schmid (1955 in press) *Stenophylax* paper was essentially completed in 1952 but not published until 1957 (see Schmid 1957). Therefore, Schmid had the *Stenophylax* information in 1955. In the 1957 (1952) paper Schmid did not mention *S.antennatus* or his 1955 placement of *S.antennatus* within *Philocasca*. This appears to indicate Schmid no longer agreed with his own 1955 placement of *S.antennatus* within *Philocasca*.

It is fascinating that this long, convoluted taxonomic history resulting in the placement of *Stenophylaxantennatus* within *Philocasca* appears to be based on the one male type from Mt. Rainier National Park, Washington, and the description of another *S.antennatus* male by Banks (1943) from Wallace, Idaho (see discussion under *M.thor* above). The Banks (1943) description of the irrorate wings, “dark crescentic lobe” dorsally on the aedeagus, and upturned spines (parameres) are clearly not characteristic of *Philocasca*.

Subsequent authors (Flint 1966, Wiggins and Anderson 1968, Wiggins 1977, Anderson et al. 1982, Wiggins 1996, Schmid 1998, etc.) followed Schmid’s 1955 placement of *Stenophylaxantennatus* within *Philocasca*, rather than Banks (1943).

Wiggins and Anderson (1968) summarized the known *Philocasca* species. They provided re-descriptions and illustrations for the holotypes of *P.demita*, *P.oron*, *P.antennata*, and *P.banksi* males. They also provided new descriptions and figures for the *P.demita* female and larva; *P.oron* female; and *P.rivularis* male, female and larvae. There is also a description for an unknown *Philocasca* larva. Additional specimens representing this unknown *Philocasca* species have been examined from Idaho and Montana and, with the association of the *Stenophylaxantennatus* larva, we conclude the unknown larva of Wiggins and Anderson (1986) is *P.banksi*.

Nimmo (1971) described *P.thor* from a single Alberta collection. The first author examined this holotype and compared it to three additional Alberta *P.thor* males in the Barcode of Life DNA voucher collection, Guelph. They are the same. The female and larvae of *P.thor* have yet to be associated.

Nimmo (1977) described *P.alba* from three collections in southern Alberta; including, as paratypes, material the second author collected.

During his studies on litter processing and insect life history within a small Alberta stream, Mutch (1981) discovered that the primary insect litter processor was *P.alba*. Mutch also noted that the larva associated with *P.alba* was not similar to the other known *Philocasca* larvae. He conducted a review of *Philocasca* (Mutch 1981) and concluded that the seven *Philocasca* species belong within two separate groups, suggesting that *P.antennata*, *P.alba*, and *P.thor* belonged within a different genus. Further studies planned at that time to resolve the issue ceased until now. The first author compared the *P.alba* holotype to material from this southern Alberta population and they are the same, resulting in associations of larvae, pupae, males and females for *P.alba*.

Schmid (1998) provided an additional description and illustrations of the *P.thor* male and the *P.demita* female.

It was not until the Blinn and Ruiter (2013) collections of *P.antennata* from Mt. Baker, Washington, that *P.antennata* material other than the holotype was located. This is likely due to the difficulty of collecting high altitude, isolated habitats early in the season. The Mt. Baker material contained males, females and associated *P.antennata* larvae and also led to the conclusion that *P.antennata*, *P.alba*, and *P.thor* did not belong within *Philocasca*.

## Supplementary Material

XML Treatment for Montiphylax

XML Treatment for Montiphylax antennatus

XML Treatment for Montiphylax thor

XML Treatment for Montiphylax albus

## Footnotes

† The larvae of Chilostigmodes remain un-described although it is expected to have single filament gill clusters. Chilostigmodes records occur throughout northern North America from Alaska to Newfoundland.
